# Anti-obesity effect of Neoagaro-oligosaccharides with overweight and obese subjects: a 16-week, randomized, double-blind, placebo-controlled clinical trial

**DOI:** 10.1186/s12906-023-04206-2

**Published:** 2023-10-19

**Authors:** Hyang-Im Baek, Ki-Chan Ha, Yu Kyung Park, Je Hyeon Lee, Eun Joo Kim, Hye-Jeong Ko, Jong Cheon Joo

**Affiliations:** 1 Department of Food Science & Nutrition, Woosuk University, Wanju, 55338 Republic of Korea; 2 Healthcare Claims & Management Inc, Jeonju, 54858 Republic of Korea; 3 Dyne Bio Inc, Sungnam, 13209 Republic of Korea; 4 Department of Sasang Constitutional Medicine, College of Korean Medicine, Wonkwang University, Iksan, 54538 Republic of Korea

**Keywords:** Neoagaro-oligosaccharides, Overweight, Obesity, Visceral fat, Body weight, Clinical trial

## Abstract

**Background:**

This trial aimed to evaluate the anti-obesity effects and safety of Neoagaro-oligosaccharides (NAOs) in humans in a 16 week, randomized, double-blind, placebo-controlled clinical trial.

**Methods:**

One hundred overweight or obese subjects with a body mass index of 23 to 34.9 kg/m^2^ and a percent body fat of > 25% for males or > 30% for females were enrolled. NAOs or placebo products were administered at 3 g (twice a day, four capsules once) each for 16 weeks. Efficacy and safety biomarkers were measured before and after intervention.

**Results:**

After 16 weeks of intervention, the group administered with NAOs had statistically significant decreases in visceral fat area and visceral-subcutaneous fat area ratio compared to the placebo group. The NAOs group suppressed the increase in weight and BMI compared to the placebo group, which was significant between groups. High-density lipoprotein- cholesterol was increased in the group administered with NAOs, which showed a significant trend compared to the placebo group. Clinical changes were not observed for any safety biomarkers.

**Conclusions:**

These results suggest that NAOs have a beneficial effect on obesity. Thus, NAOs could be used as an anti-obesity supplement without side effects.

**Trial registration:**

cris.nih.go.kr: (KCT0006640, 07/10/2021).

## Background

The prevalence of overweight and obesity has increased globally, especially in developed countries [1]. According to the World Health Organization (WHO), in 2016 more than 1.9 billion adults (18 years and older) were overweight, and about 650 million were obese with 39% of the world’s population being overweight or obese [2]. Given the current trend, it has been predicted that half of the world’s adult population will be overweight or obese by 2030 [3]. Therefore, obesity is a serious worldwide health problem.

The cause of obesity is highly complex. It includes genetic, physiological, environmental, social, and economic factors and a high energy intake relative to energy expenditure, the most common cause of obesity [4]. Overweight and obesity characteristically accumulate body fat for various causes, resulting in weight gain. Obesity is not only a cosmetic problem, but also a health threat that can increase the occurrence of metabolic diseases such as type 2 diabetes mellitus (T2DM) associated with insulin resistance, cardiovascular disease (CVD), hyperlipidemia, fatty liver disease, and several types of cancers [5, 6]. Overweight and obesity are also associated with decreased quality of life [7, 8] and increased morbidity and mortality [9]. Consequently, obesity has increased the economic burden on the health care system due to hospitalizations and drug prescriptions for obesity-related diseases [10]. Therefore, it is necessary to develop health functional foods with an obesity preventive effect without side effects.

*Gelidium elegans (G. elegans)* is an edible red algae native to Asia. It has been safely consumed for a long time [11]. Agar is a major component of the cell wall of red algae. In Asia, agar has long been recognized as generally safe. Due to its unique gel-forming physicochemical properties, it has been used as a food additive and gelling agent in puddings, jellies, and other confectionery [12]. Agar is a heterogeneous polysaccharide composed of repeating units of β-1,4-D-galactopyranosyl-α-1,3-L-galactopyranose [13]. Neoagaro-oligosaccharides (NAOs) are made by hydrolyzing agar/agarose and are prepared by breaking β-(1–4) bonds using β-agarase [14, 15]. NAOs have various activities, including anti-obesity [13–16], hypolipidemic [17], antioxidant [18–21], anti-inflammatory [22–24], and whitening of melanoma cells [25, 26] effects.

In previous preclinical studies, anti-obesity effects of NAOs have been reported, including weight loss, adipocyte size reduction, and blood glucose and lipid improvement [13, 14, 17]. In addition, NAOs supplementation in obese mice improved body weight and metabolic syndrome by increasing the abundance of intestinal microorganisms such as *Eubacterium fissicatena* and Ruminococcaceae UCG-005 and showed anti-obesity effects [15].

However, no evidence has been found in humans for the anti-obesity effects of NAOs. Therefore, the objective of the present study was to evaluate the anti-obesity efficacy and safety of NAOs intake for 16 weeks by conducting a randomized, double-blind, placebo-controlled clinical trial in overweight and obese adults.

## Methods

### Study design

This 16-week, single-center, randomized, double-blind, placebo-controlled, parallel-group, clinical study was conducted at Wonkwang University Korean Medicine Hospital in Jeonju from August 2021 to May 2022. The study protocol and informed consent form were approved by the Institutional Review Board (IRB) of Wonkwang University Korean Medicine Hospital (IRB approval No.: WUJKMH-IRB-2021-0008). This study was registered with the Clinical Research Information Service (CRIS), the Republic of Korea (http://cris.nih.go.kr. clinical trial No.: KCT0006640, registered on 07/10/2021). This study was performed in accordance with the Declaration of Helsinki and the Guideline for Good Clinical Practice by the International Conference on Harmonization (ICH GCP). All participants provided written informed consent before participating in this study.

Participants were recruited through several ways (banner, newspaper, Wonkwang University Korean Medicine Hospital web page, and so on). A screening visit was conducted within two weeks of the first visit to select subjects who met the inclusion & exclusion criteria. After baseline evaluation, participants were randomly assigned to test and placebo groups at a 1:1 ratio. One hundred subjects received each product at each visit every eight weeks for three visits (visit 1: week 0; visit 2: week 8; and visit 3: week 16).

During the 16-week study period, subjects were asked to maintain their usual lifestyle including dietary intake and physical activity. They were asked to avoid consuming other functional foods or dietary supplements. Efficacy and safety were evaluated before and after the study period.

### Study subjects

One hundred participants were eligible after screening tests such as questionnaires, physical examinations, and laboratory examinations. They were enrolled within two weeks after providing informed consent and before they were given supplement.

Inclusion criteria were as follows: (1) those aged between 19 and 65 years old on screening test; (2) those with a body mass index (BMI) ≤ 34.9 kg/m² but ≥ 23 kg/m², percent body fat (PBF) > 25% for males and PBF > 30% for females; and (3) those who provided written consent after being thoroughly educated about the study’s aims and goals.

Exclusion criteria were as follows: (1) subjects with a weight change of 10% or more within 3 months prior to the screening test; (2) those who took supplement of medicines or health functional foods that might affect body weight within 1 month prior to the screening test; (3) subjects who had an obesity surgery within 1 year; (4) those with a clinically acute disease or chronic cardiovascular, endocrine, immune, respiratory, hepatobiliary, kidney, urinary, neuropsychiatric, musculoskeletal, inflammatory, hematological, or gastrointestinal disease; (5) diabetic patients who were treated with oral hypoglycemic agents or insulin on screening tests; (6) those with a history of clinically significant hypersensitivity to seaweed or agar; (7) those who were undergoing fasting therapies; (8) those who were administered with antipsychotics within 3 months prior to the screening test; (9) those with a history of medicine abuse; (10) those who participated in other human tests within three months prior to the screening test; 11) those who had the following diagnostic and medical test results: ☞ aspartate transaminase (AST) or alanine transaminase (ALT) > 3 times the upper limit of the reference range; ☞ serum creatinine > 2.0 mg/dL; 12) those who were pregnant or nursing; 13) subjects within 6 months of childbirth; 14) those who were fertile and not taking contraceptives; and 15) those who were judged by the principal investigator to be inappropriate for participation in this study because of laboratory test results and so on.

### Study products

**Fig. 1:**
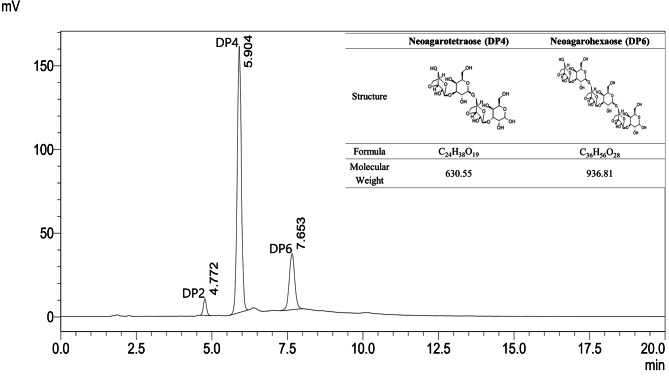
Representative chromatograms of NAOs based on high-performance liquid chromatography (HPLC) analysis of Neoagarotetraose (DP4) and Neoagarohexaose (DP6)

Test products used in this study were provided by Dyne Bio Inc.(Sungnam, Korea). As described in previous studies [13–15, 17], NAOs were prepared by dissolving *G. elegans* into agar, enzymatically reacted with β-agarase, concentrated, and dried. NAOs are produced from agar by cleavage of the β-1,4 bond by β-agarase. For standardization of NAOs, Neoagarotetraose (DP4) and Neoagarohexaose (DP6) were set as standard compounds, and were standardized to 280.05 mg/g and 197.48 mg/g, respectively. A high-performance liquid chromatography (HPLC) chromatogram of the NAOs is shown in Fig. 1.

The 3 g/day dose is based on our preclinical studies [13], that including the same dose significantly reduced weight in the obese mouse model. In addition, the intake of powdered agar, a notified health functional food (HFF) approved by Korea’s Ministry of Food and Drug Safety of Korea (MFDS) for improving bowel movements, is 2 to 5 g/day, and the intake of extract of *G. elegans*, an individually approved HFF for weight loss, is 1 g/day. Therefore, the average animal test and HFF intake is equivalent to 3 g/day.

All subjects were randomly assigned to a group administered with NAOs (3 g/day of NAOs) or a placebo group (0 g/day of NAOs). Subjects took 3 g per day (twice after breakfast and dinner, four capsules once) for 16 weeks. The placebo capsules contained corn starch with the same weight, energy, carbohydrate content and appearance as the test capsules (Table 1).

**Table 1:** Composition of the test products

Component	Content (%)	
	NAOs group	Placebo group
NAOs (Neoagaro-oligosaccharides)	100	
Corn starch		100
Total	100.0	100.0

NAOs, Neoagaro-oligosaccharides

### Efficacy outcome measurements

All subjects were subjected to efficacy evaluation before and after intake for 16 weeks during the study period. To measure abdominal fat area, subjects underwent a computed tomography (CT; WCT-200-140, Hispeed Dual) scan. Ab-dominal fat CT scans were taken with subjects lying on their backs. Total abdominal fat area (TFA), visceral fat area (VFA), and subcutaneous fat area (SFA) were measured, and the visceral-subcutaneous fat area ratio (VSR) was calculated. The same machine was used throughout the study period to evaluate changes in anthropometric parameters, including body weight, body mass index (BMI; kg/m^2^), waist circumference (WC), hip circumference (HC), and waist-hip ratio (WHR). Body fat mass (BFM), percent body fat (PBF), and lean body mass (LBM) were analyzed using dual-energy X-ray absorptiometry (DEXA; Primus, OsteoSys). Blood collection was performed in a fasting state for lipid profiles assessment, including total cholesterol, low-density lipoprotein-cholesterol (LDL-C), high-density lipoprotein-cholesterol (HDL-C), triglyceride, and free fatty acid.

### Safety Outcome measurements

To evaluate safety, all adverse events (AEs) were monitored. Laboratory tests, urinalysis, vital signs, and electrocardiograms were performed before and after 16 weeks of administration. Laboratory tests included hematological tests [white blood cell (WBC), red blood cell (RBC), hemoglobin, hematocrit, platelets] and blood bio-chemical tests [alkaline phosphatase (ALP), AST, ALT, total bilirubin, total protein, albumin, gamma-glutamyl transferase (gamma-GT), blood urea nitrogen (BUN), creatinine, glucose, lactate dehy-drogenase (LD), creatine kinase (CK), high sensitivity C-reactive protein (hs-CRP)]. Blood was collected after fasting for 12 h. Urinalysis measures specific gravity and pH in urine. Systolic blood pressure (SBP), diastolic blood pressure (DBP), and pulse were measured to evaluate vital signs.

### Dietary intake and physical activity assessment

During the study period, subjects maintained their usual dietary intake. Changes in dietary intake were evaluated through a standardized 3-day dietary record (2 days on weekdays, 1 day on weekends). Dietary intake was analyzed by a trained dietitian. Total daily intakes of energy, carbohydrates, lipids, protein, and fiber were analyzed using a Computer-Aided Nutritional analysis program (CAN-pro, Korean Nutrition Society, Seoul).

Subjects were asked to maintain their usual physical activity during the study period. Changes in physical activity were measured according to activity using a global physical activity questionnaire (GPAQ). GPAQ is a questionnaire that measures physical activity in three domains, including work (vigorous-to-moderate intensity activity for more than 10 min for daily work-related activities), transport (activities such as cycling and walking for more than 10 min for movement of place), and recreation (vigorous-to-moderate intensity activity for more than 10 min for leisure activities) [27]. Metabolic equivalent of task (MET) values were calculated using GPAQ data.

### Statistical analysis

The sample size was referenced from a similar previous study [28]. It was determined to achieve 80% statistical power with an alpha of 0.05. The sample size for each group was determined by allowing a dropout rate of 20%. Based on the calculation, 50 subjects per group (a total of 100 subjects) were needed.

All statistical analyses were performed using SAS® (version 9.4; SAS Institute, Cary, NC, USA). Continuous variables were presented as means ± standard deviation (SD) and categorical variables were presented as frequencies. Data analysis for efficacy was performed using the full analysis set (FAS). Analysis for safety was performed using the safety set. Statistical analysis was performed for data according to protocol criteria. Significant differences in demographic characteristics at baseline were analyzed using the Chi-square test (Fisher’s exact test). Mean comparisons between the two groups were performed using an independent t-test. Statistical analysis between the two groups was performed using an independent t-test for change values before and after 16 weeks of intervention. Within each group, the comparison between before intake and 16 weeks after intake was analyzed using a paired t-test. Differences were considered statistically significant at a p-value < 0.05.

## Results

### Baseline characteristics of subjects

**Fig. 2:**
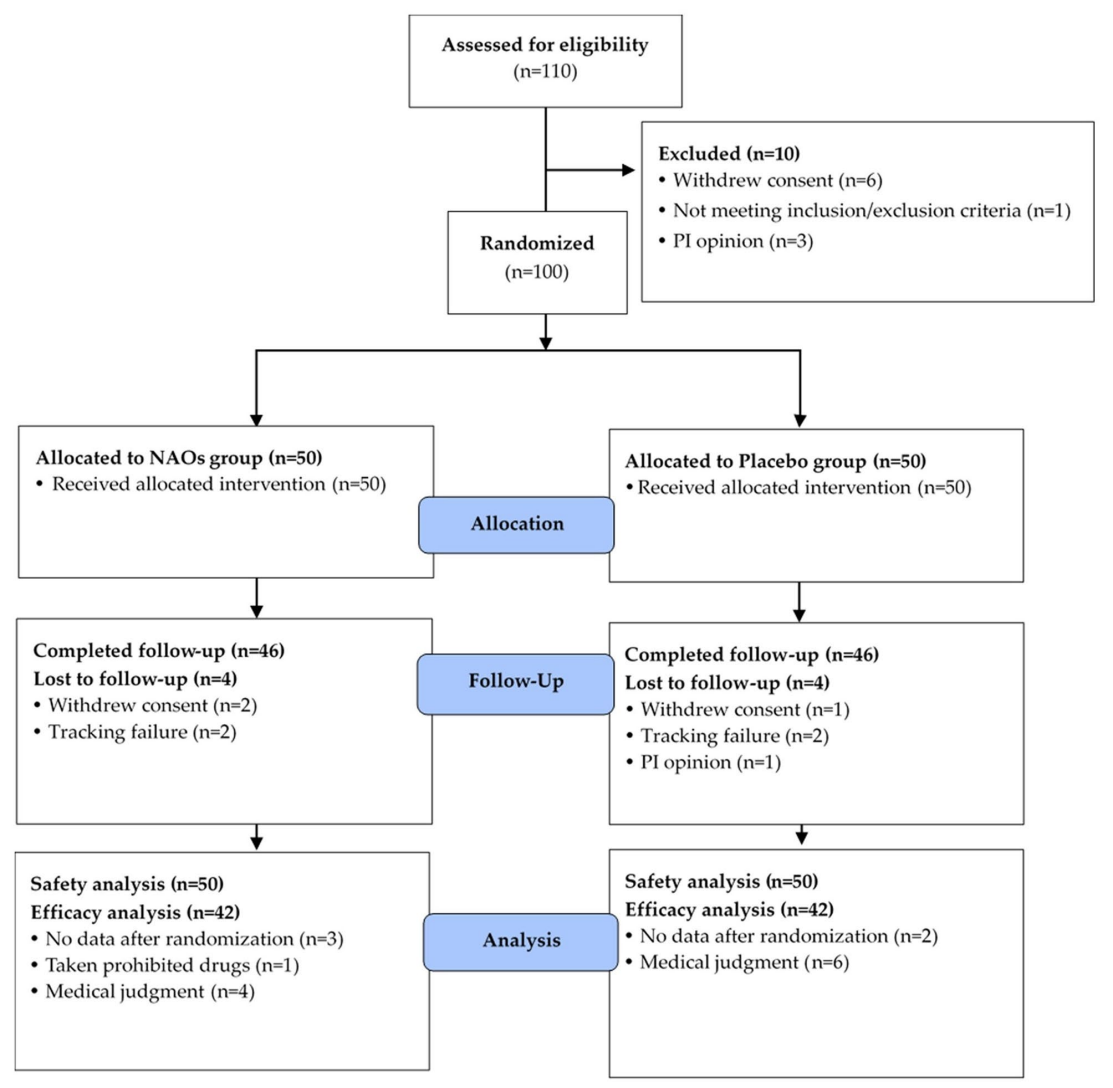
Flow chart of subjects. Number of study participants enrolled, allocated, followed, and analyzed, shown using the CONSORT 2010 Flow Diagram. FAS, full analysis set

A total of 110 participants were screened, and 100 subjects who met the inclusion/exclusion criteria were selected and randomly assigned to the NAOs and placebo groups. A total of 84 subjects were examined at follow-up according to the criteria of protocol (Fig. 2). Demographic characteristics measured at baseline of 84 subjects have been summarized in Table 2. There were no significant differences in baseline characteristics such as gender, age, height, weight, BMI, PBF, SBP, DBP, or pulse measured between the two groups at the screening visit or visit 1.

**Table 2:** Baseline demographic characteristics of subjects

Variable	NAOs group (n = 42)	Placebo group (n = 42)	Total (n = 84)	*p*-value^1)^
Sex (M/F)	0/42	0/42	0/84	
Age (years)	36.405 ± 10.258	37.405 ± 9.515	36.905 ± 9.847	0.645
Height (cm)	160.000 ± 5.622	162.071 ± 6.264	161.036 ± 6.007	0.115
Weight (kg)	67.436 ± 6.917	68.690 ± 7.578	68.063 ± 7.239	0.430
BMI (kg/m^2^)	26.324 ± 2.139	25.963 ± 1.874	26.146 ± 2.009	0.417
PBF (%)	36.933 ± 3.809	36.533 ± 3.243	36.733 ± 3.522	0.606
SBP (mmHg)	122.810 ± 10.530	119.786 ± 9.473	121.298 ± 10.071	0.170
DBP (mmHg)	75.476 ± 9.505	73.214 ± 9.188	74.345 ± 9.361	0.271
Pulse (beats/minute)	78.833 ± 10.329	76.452 ± 8.746	77.643 ± 9.587	0.258
TSH (µIU/mL)	2.148 ± 0.806	2.362 ± 1.050	2.255 ± 0.936	0.297
Alcohol (n, %)	22 (52.38)	17 (40.48)	39 (46.43)	0.274^2)^
Alcohol (units/week)	5.173 ± 5.328	3.371 ± 2.310	4.387 ± 4.331	0.165
Smoking (n, %)	0 (0.00)	0 (0.00)	0 (0.00)	

NAOs, Neoagaro-oligosaccharides; BMI, body mass index; PBF, percent body fat; SBP, systolic blood pressure; DBP, diastolic blood pressure; TSH, thyroid-stimulating hormone

Values are presented as mean ± SD or number (%)

^1)^ Analyzed by independent t-test between the groups

^2)^ Analyzed by chi-square test between the groups

### Dietary intake and physical activity

Dietary intake surveys and physical activity questionnaires were used before and after the intervention. The results of the changes have been presented in Table 3. There were no significant differences in changes of energy, carbohydrate, lipid, protein, or fiber intake between NAOs and placebo groups.

**Table 3:** Dietary intake and Physical Activity changes

Measurements	NAOs group (n = 42)			Placebo group (n = 42)			*p*-value^2)^
	Baseline	16 week	*p*-value^1)^	Baseline	16 week	*p*-value^1)^	
Energy (kcal)	1437.869 ± 501.638	1413.147 ± 493.146	0.725	1393.406 ± 323.663	1405.211 ± 284.334	0.810	0.669
Carbohydrates (g)	199.292 ± 60.685	196.488 ± 68.177	0.791	194.786 ± 53.173	194.846 ± 40.550	0.995	0.837
Lipids (g)	45.363 ± 31.584	43.578 ± 25.586	0.538	42.155 ± 16.131	43.957 ± 14.856	0.437	0.332
Protein (g)	55.972 ± 20.505	57.616 ± 24.497	0.572	57.354 ± 14.531	56.459 ± 16.431	0.725	0.510
Fiber (g)	14.119 ± 8.151	13.423 ± 5.880	0.389	14.466 ± 6.002	15.193 ± 6.250	0.417	0.237
MET (min/week)	1748.190 ± 4727.484	1037.143 ± 2003.833	0.188	1223.810 ± 1805.402	1051.429 ± 2721.270	0.722	0.454

NAOs, Neoagaro-oligosaccharides

Values are presented as the mean ± SD

^1)^ Analyzed by paired t-test between baseline and 16 weeks within each group (Weeks 0 vs. 16)

^2)^ Analyzed by independent t-test for change value between the groups (NAOs vs. Placebo)

There were no significant differences in MET values, indicating similar physical activity in the two groups. Therefore, dietary intake and physical activity during the study period were well maintained without affecting study results.

### Efficacy evaluation

**Fig. 3:**
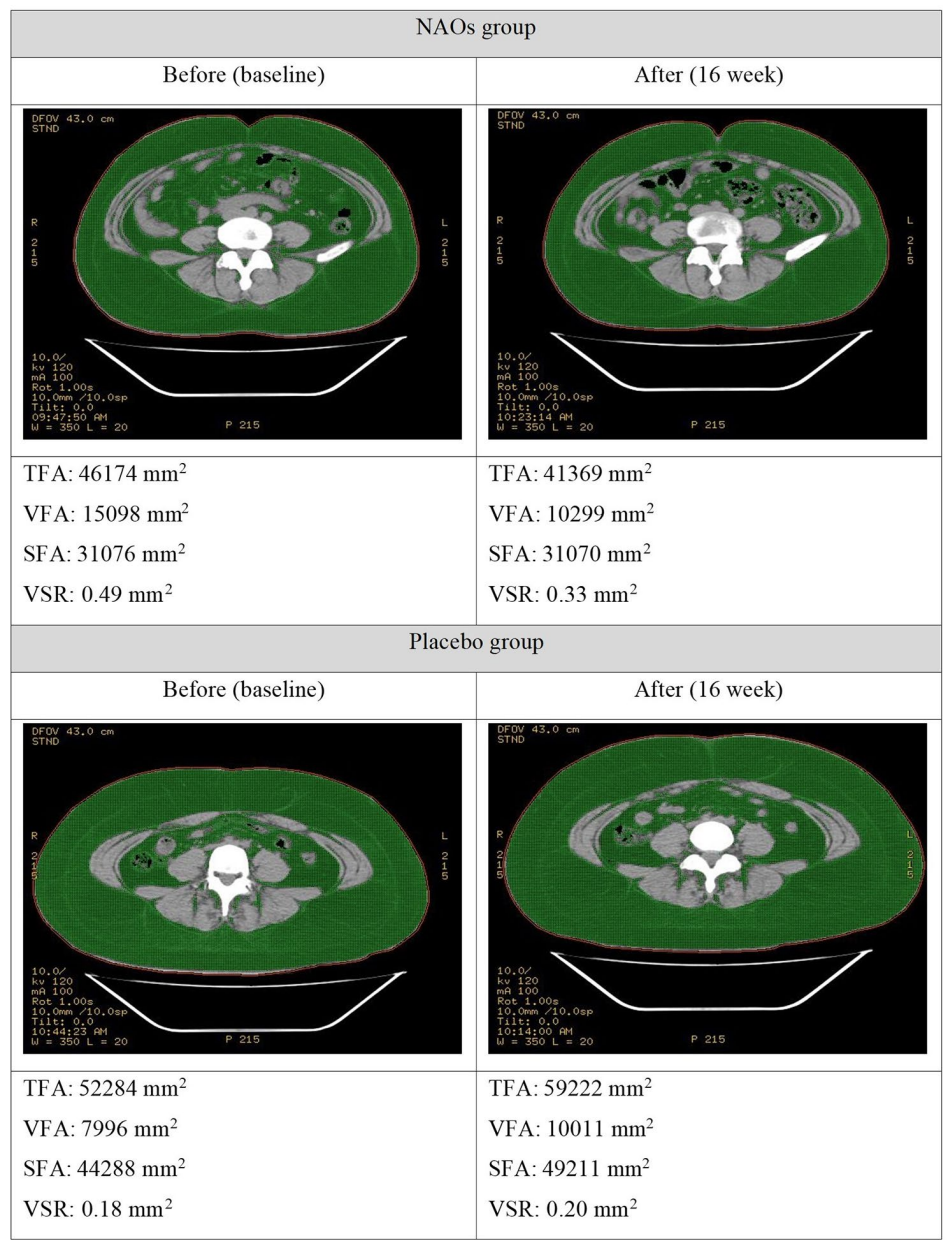
Representative abdominal fat CT data at baseline and 16 weeks in a subject from NAOs or placebo group. NAOs, Neoagaro-oligosaccharides; CT, computed tomography; TFA, total abdominal fat area; VFA, visceral fat area; SFA, subcutaneous fat area; VSR, visceral-subcutaneous fat area ratio

**Fig. 4:**
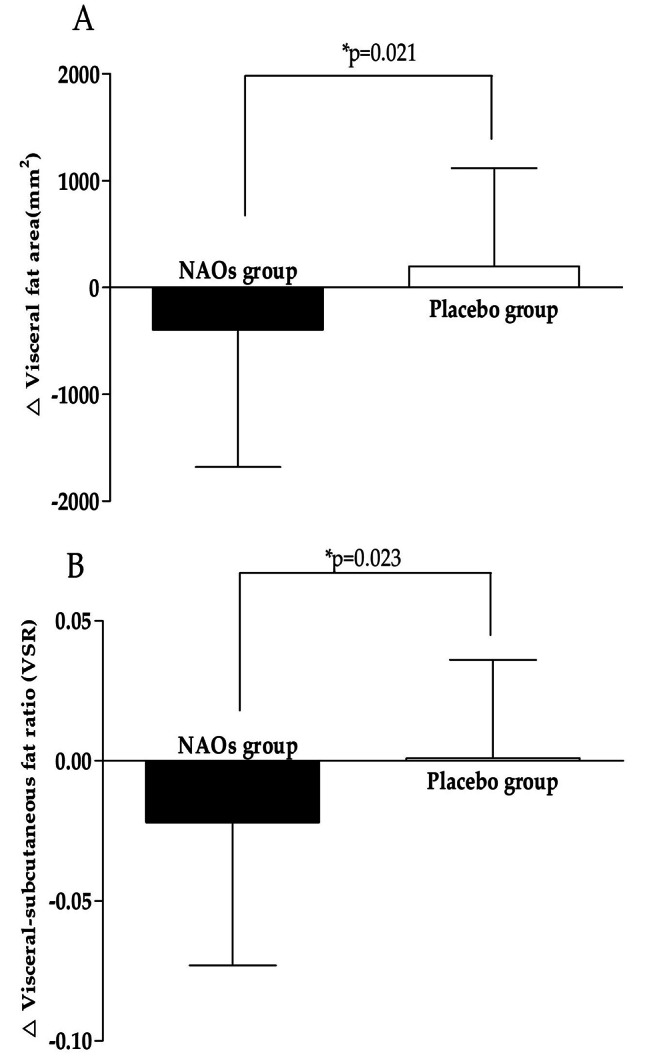
Change in body fat areas. (A) Visceral fat area(VFA), (B) Visceral-subcutaneous fat ratio(VSR) were measured in NAOs and placebo treated groups at baseline and 16 weeks. Values are pre-sented as mean ± SD. Between-group differences were assessed using independent t-test for change value between the groups. *p < 0.05 vs. placebo group. NAOs, Neoagaro-oligosaccharides; VFA, visceral fat area; VSR, visceral-subcutaneous fat area ratio

**Fig. 5:**
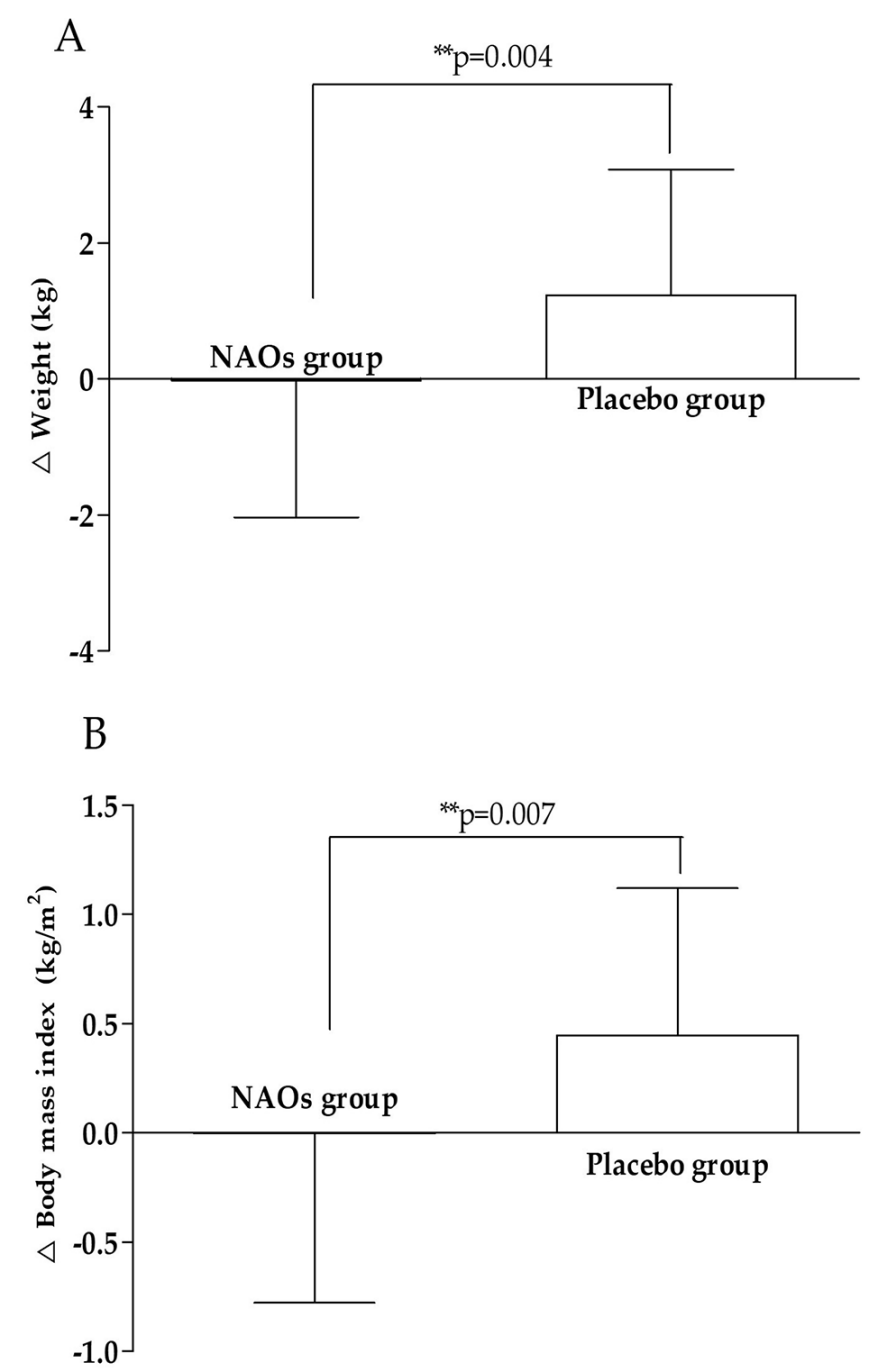
Change in anthropometric parameters. (A) Weight, (B) Body mass index (BMI) were measured in NAOs and placebo treated groups at baseline and 16 weeks. Values are presented as mean ± SD. Between-group differences were assessed using independent t-test for change value between the groups. **p < 0.01 vs. placebo group. NAOs, Neoagaro-oligosaccharides; BMI, body mass index

Efficacy evaluation biomarkers were measured before intervention and 16 weeks after intervention. Results are shown in Table 4; Fig. 3, 4 and 5. The change value of VFA was − 393.975 ± 1288.335 mm^2^ in the group administered with NAOs and + 198.425 ± 918.599 mm^2^ in the placebo group, showing a statistically significant difference between the two groups (*p* = 0.021). The VSR in the NAOs group changed to -0.022 ± 0.051 after 16 weeks of intake compared to that before intake. It was significantly reduced within the group (*p* = 0.011). The change was significantly different compared to the placebo group (*p* = 0.023).

**Table 4:** Changes in efficacy outcomes before and after 16 weeks of intake

Measurements	NAOs group (n = 42)			Placebo group (n = 42)			*p*-value^2)^
	Baseline	16 week	*p*-value^1)^	Baseline	16 week	*p*-value^1)^	
**Abdominal fat CT**							
TFA (mm^2^)	33826.214 ± 8327.489	34069.476 ± 8959.364	0.557	33894.190 ± 6839.269	34558.452 ± 7820.549	0.105	0.465
VFA (mm^2^)	7860.400 ± 3268.930	7466.425 ± 3233.311	0.060	8003.375 ± 2823.643	8201.800 ± 2996.630	0.180	**0.021** ^*^
SFA (mm^2^)	25325.738 ± 6121.032	25869.095 ± 6777.136	0.114	24966.317 ± 5861.417	25384.927 ± 6208.211	0.177	0.785
VSR	0.316 ± 0.123	0.294 ± 0.121	**0.011** ^*^	0.340 ± 0.153	0.341 ± 0.152	0.826	**0.023** ^*^
**Anthropometric parameters**							
Weight (kg)	67.436 ± 6.917	67.412 ± 7.272	0.939	68.690 ± 7.578	69.917 ± 8.267	**0.0001** ^***^	**0.004** ^**^
BMI (kg/m^2^)	26.324 ± 2.139	26.321 ± 2.383	0.984	25.963 ± 1.874	26.407 ± 2.124	**0.0001** ^***^	**0.007** ^**^
WC (cm)	86.881 ± 6.663	86.895 ± 7.244	0.975	87.526 ± 7.074	88.388 ± 7.462	0.054	0.177
HC (cm)	99.652 ± 3.714	99.712 ± 3.527	0.876	100.712 ± 4.703	101.164 ± 5.322	0.135	0.417
WHR	0.876 ± 0.054	0.875 ± 0.054	0.957	0.880 ± 0.051	0.885 ± 0.047	0.218	0.426
**DEXA**							
BFM (g)	22478.357 ± 4305.502	23066.833 ± 4784.854	**0.032** ^*^	22449.200 ± 3663.866	23421.650 ± 3967.897	**0.001** ^**^	0.324
PBF (%)	33.969 ± 4.316	34.567 ± 4.747	**0.038** ^*^	33.921 ± 3.346	34.838 ± 3.864	**0.002** ^**^	0.414
LBM (g)	43438.810 ± 4606.042	43294.143 ± 4496.329	0.393	44408.429 ± 4276.123	44644.548 ± 4460.618	0.114	0.091
**Lipid profiles**							
Total cholesterol (mg/dL)	204.381 ± 32.628	207.048 ± 27.304	0.421	199.024 ± 35.771	194.333 ± 30.920	0.177	0.124
LDL-C (mg/dL)	125.857 ± 30.608	133.310 ± 30.205	**0.013** ^*^	118.190 ± 30.560	116.619 ± 24.765	0.567	**0.025** ^*^
HDL-C (mg/dL)	58.195 ± 10.378	60.098 ± 10.712	0.093	57.439 ± 10.943	56.415 ± 11.709	0.430	0.088
Triglyceride (mg/dL)	103.714 ± 54.035	100.571 ± 44.591	0.637	104.077 ± 47.473	114.846 ± 63.831	0.218	0.200

NAOs, Neoagaro-oligosaccharides; CT, computed tomography; TFA, total abdominal fat area; VFA, visceral fat area; SFA, subcutaneous fat area; VSR, visceral-subcutaneous fat area ratio; BMI, body mass index; WC, waist circumference; HC, hip circumference; WHR, waist-hip ratio; DEXA, dual-energy X-ray absorptiometry; BFM, Body fat mass; PBF, percent body fat; LBM, lean body mass; LDL-C, low-density lipoprotein- cholesterol; HDL-C, high-density lipoprotein- cholesterol

Values are presented as the mean ± SD

^1)^ Analyzed by paired t-test between baseline and 16 weeks within each group (Weeks 0 vs. 16)

^2)^ Analyzed by independent t-test for change value between the groups (NAOs vs. Placebo)

^*^p < 0.05, ^**^p < 0.01, ^***^p < 0.001

Among anthropometric indicators, weight was significantly increased by + 1.226 ± 1.853 kg in the placebo group (*p* = 0.0001) but decreased by -0.024 ± 2.012 kg in the NAOs group, which was statistically significant between the two groups (*p* = 0.004) as weight gain was suppressed by NAOs intake. BMI as an indicator of obesity was also significantly increased in the placebo group by + 0.444 ± 0.676 kg/m^2^ (*p* = 0.0001) but decreased by -0.002 ± 0.776 kg/m^2^ in the NAOs group, showing a statistically significant difference between NAOs and placebo groups (*p* = 0.007) as the increase in BMI was suppressed by NAOs intake.

Regarding blood lipid profile, the HDL-C value was increased by + 1.902 ± 7.074 mg/dL in the NAOs group but decreased by -1.024 ± 8.226 mg/dL in the placebo group, showing a trend of significant difference between the two groups (*p* = 0.088). In addition, there was no significant difference in the efficacy evaluation index.

### Safety evaluation

During the study period, 19 cases (11 cases in the NAOs group and 8 cases in the placebo group) showed mild adverse reactions. The adverse reactions in the NAOs group were 1 case of sore throat, 1 case of cold (sore throat), and 9 cases of COVID-19. In the placebo group, there was 1 case of dyspepsia, 1 case of allergic rhinitis, 1 case of back pain, and 5 cases of COVID-19. However, the incidence of adverse events showed no statistically significant difference between the two groups (*p* > 0.05). All adverse events were not clearly related to the intake of the investigational product.

The two groups had no significant differences in the results of blood tests, electrocardiograms, or vital signs for safety evaluation. They were all within their normal ranges. During this study, no clinically significant changes were observed (Table 5). Thus, intake of NAOs was evaluated as safe for humans.

**Table 5:** Changes in safety outcomes before and after 16 weeks of intake

Measurements	NAOs group (n = 50)			Placebo group (n = 50)			*p*-value^2)^
	Baseline	16 week	*p*-value^1)^	Baseline	16 week	*p*-value^1)^	
ALP (IU/L)	181.960 ± 46.979	175.780 ± 44.001	**0.047** ^*^	172.140 ± 51.286	163.500 ± 35.514	0.184	0.730
AST (IU/L)	21.560 ± 7.754	22.240 ± 9.544	0.690	20.560 ± 6.168	21.320 ± 9.153	0.556	0.970
ALT (IU/L)	18.920 ± 9.733	21.420 ± 14.058	0.189	20.760 ± 11.559	22.320 ± 17.808	0.525	0.760
Total bilirubin (mg/dL)	0.631 ± 0.249	0.641 ± 0.249	0.730	0.643 ± 0.270	0.590 ± 0.225	0.113	0.153
Total protein (g/dL)	7.150 ± 0.380	6.974 ± 0.416	**0.002** ^**^	7.114 ± 0.430	7.000 ± 0.368	**0.028** ^*^	0.407
Albumin (g/dL)	4.322 ± 0.267	4.270 ± 0.293	0.167	4.316 ± 0.223	4.274 ± 0.211	0.075	0.819
gamma-GT (IU/L)	20.200 ± 11.019	21.700 ± 15.360	0.213	19.220 ± 8.092	22.280 ± 19.461	0.247	0.589
BUN (mg/dL)	11.838 ± 3.665	11.970 ± 3.039	0.755	11.338 ± 3.270	11.500 ± 3.333	0.740	0.963
Creatinine (mg/dL)	0.768 ± 0.117	0.772 ± 0.105	0.766	0.776 ± 0.094	0.760 ± 0.076	0.221	0.285
Glucose (mg/dL)	96.800 ± 7.478	96.160 ± 9.498	0.535	95.360 ± 9.672	95.880 ± 8.578	0.682	0.477
LD (IU/L)	170.860 ± 31.504	173.000 ± 29.956	0.548	168.220 ± 25.471	167.200 ± 22.257	0.755	0.512
CK (IU/L)	157.560 ± 494.237	144.360 ± 363.930	0.879	80.440 ± 34.283	91.520 ± 47.675	0.156	0.784
hs-CRP (mg/L)	1.600 ± 2.330	1.346 ± 1.391	0.324	0.920 ± 0.971	0.948 ± 1.217	0.854	0.345

NAOs, Neoagaro-oligosaccharides; ALP, alkaline phosphatase; AST, aspartate aminotransferase; ALT, alanine aminotrans-ferase; gamma-GT, gamma-glutamyl transferase; BUN, blood urea nitrogen; LD, lactate dehydrogenase; CK, creatine kinase; hs-CRP, high sensitivity C-reactive protein

Values are presented as the mean ± SD

^1)^ Analyzed by paired t-test between baseline and 16 weeks within each group (Weeks 0 vs. 16)

^2)^ Analyzed by independent t-test for change value between the groups (NAOs vs. Placebo)

^*^p < 0.05, ^**^p < 0.01

## Discussion

We performed a 16-week, randomized, double-blind, placebo-controlled clinical trial to evaluate the anti-obesity efficacy and safety of NAOs in overweight and obese subjects. After 16 weeks of intake, VFA and VSR were significantly reduced in the NAOs group, and weight and BMI were significantly suppressed from increase compared to the placebo group. HDL-C showed a tendency to increase significantly in the NAOs group compared to the placebo group.

Obesity is defined as an excess of body fat [29]. The association of obesity, insulin resistance, and chronic low-grade inflammation has been evident for several years [30]. It is related to the occurrence of metabolic diseases such as CVD and diabetes [31, 32]. The importance of abdominal visceral fat in maintaining health, makes accurate visceral fat measurement crucial. Among the methods for measuring abdominal visceral fat, waist circumference (WC) is an easy to measure indicator of obesity [32, 33]. While, measurement of visceral fat and subcutaneous fat by computed tomography (CT) can more accurately reflect the amount of visceral fat and risks of various metabolic diseases and cardiovascular disease, it is expensive and risks radiation exposure [34, 35]. The VSR along with absolute fat mass or area is an indicator of body fat distribution that correlates with CVD risk [36]. Indeed, the relative distribution of abdominal fat might be more important than visceral and subcutaneous fat areas as VSR is more strongly associated with risk of CVD than visceral fat area [36]. In this study, intake of NAOs significantly reduced VFA and VSR compared to the intake of placebo. These results suggest that NAOs can reduce body fat accumulation, and prevent the progression of metabolic syndrome caused by obesity.

BMI has long been recognized as a predictor of morbidity and mortality from numerous chronic diseases, including type 2 diabetes, CVD, and stroke [37, 38]. Recent clinical guidelines indicate that BMI best classifies disease risk and that obesity can be diagnosed based on BMI. In Asia, overweight, obesity grade I, and obesity grade II are defined as body mass index (BMI) of 23–24.9 kg/m^2^, 25–29.9 kg/m^2^, and > 30 kg/m^2^, respectively [39]. This study set a BMI of 23 to 35 kg/m^2^ as the selection criterion for overweight and obese subjects. Those with severe obesity requiring treatment with a BMI > 35 kg/m^2^ were excluded. The results of NAOs intake for 16 weeks confirmed that the increase in body weight and BMI was suppressed.

The mechanism of the anti-obesity effect of NAOs was confirmed in several preclinical studies, including weight loss, adipocyte size reduction, blood glucose and lipid improvement through PPAR-r and MAPK signaling pathways, adiponectin regulation, and gut microbiome modulation [13–15, 17]. The effect of NAOs on HFD-induced obese mice effectively suppressed obesity and metabolic diseases associated with obesity (hyperlipidemia, steatosis, insulin resistance, and glucose intolerance) through increased adiponectin production and regulation of the gut microbiome. [13, 15]. In mice with type 2 diabetes induced by a high-fat diet and injection of alloxan, NAOs had the effect of improving lipid metabolism and lipid accumulation through regulation of MAPK-Nrf2 and PPARγ pathway [14]. Therefore, the intake of NAOs in humans was consistent with the results of preclinical tests conducted with the same raw materials, confirming the consistency of the study results.

This study found no differences in biomarkers except VFA, VSR, weight, and BMI. Obesity is influenced by various factors, including individual characteristics, dietary intake, and physical activity [40]. Additionally, it can be difficult to interpret observed differences between intervention groups in weight management RCTs. Because intercurrent events that occur after study initiation may affect the interpretation of results at the end of the intervention [41]. Therefore, more accurate studies are required to detect the variations in other indicators of obesity and to find precise mechanisms under the control of lifestyle factors, food intake and physical activity. This study was conducted from August 2021 to May 2022 and was conducted during the COVID-19 pandemic. The global lockdown restrictions to curb the COVID-19 pandemic have altered many aspects of daily life, including diet and physical activity [42]. Nevertheless, VFA, VSR, weight, and BMI were significantly changed by NAOs intake compared to the placebo group, confirming the anti-obesity effect.

Placebo is used to control for placebo effects in randomized clinical trials (RCTs) because it is necessary to blind researchers or subjects to prevent influence on study results. A placebo looks like a test product but contains no active ingredients. Placebos must have the same appearance, same dosage, same delivery method, and be taken at the same time at the same frequency [43]. Therefore, in this study, corn starch, which had similar weight, appearance, calories, and carbohydrate content to the test product and had no functions or side effects, was used as a placebo ingredient. For this reason, corn starch is used as a placebo ingredient in several clinical studies [44–46]. Therefore, we believe that corn starch, used as a placebo ingredient in this study, would have had a weak effect on the results.

In preclinical studies, NAOs decreased total cholesterol, triglyceride, and LDL-C and increased HDL-C in the blood of animal models [14, 15, 17]. Because NAOs increased the nuclear translocation of SREBP-2 and showed a lipid improvement effect by regulating a crucial transcription factor for LDLR gene expression [17]. However, in this clinical study, LDL-C and HDL-C increased or tended to increase, and total cholesterol and triglyceride did not change. All biomarkers were within the normal range, and no clinically significant changes were observed, so it was evaluated as safe with no effect on lipid metabolism. The baseline values of lipid profiles in Table 4 indicate that the average lipid profile in this study was within the normal range. The subjects recruited in this study were overweight or obese but were healthy without any signs of metabolic syndrome or dyslipidemia. Therefore, it was evaluated as safe for healthy people to consume NAOs without clinical problems. Additionally, NAOs have been recognized as a New Dietary Ingredient (NDI) by the U.S. FDA (NDI Number: NDI 1237) and as other food processed products by Korea’s MFDS (Item Manufacturing Report No.: 19960372607460) and can be used as food raw materials. Therefore, NAOs have been recognized as safe. In this present study, clinically meaningful adverse reactions or body changes (blood test, electrocardiogram or vital signs) were not observed during the clinical trial, indicating that the consumption of NAOs was safe for humans.

In this clinical trial, no side effects or serious adverse reactions were observed in blood tests, electrocardiograms, or vital signs, or reported in interviews. These results confirm that daily intake of NAO for 16 weeks is safe for humans.

Our study is the first human study about the anti-obesity effect of NAOs. However, a few limitations of this study should be considered. First, although the number of subjects was calculated by referring to previous similar studies, the sample size was small, limiting our findings’ generalizability. Large-scale studies are needed in the future. Second, regarding the gender of subjects, all subjects in this study were women. It is generally known that the body fat percentage and incidence of obesity are higher in women than in men [47, 48]. For this reason, female subjects were mainly recruited and participated. However, to generalize the effects of NAOs, it is necessary to consider gender. Thus, men should be included in such studies in the future. Third, questionnaires on bowel habits were not measured, and no stool samples were collected for gut microbiome. It is known that the gut microbiome plays a crucial role in the development of obesity [49, 50]. In a previous study, NAOs showed anti-obesity effects through gut microbiome modulation in obese rats [15]. Additionally, clinical studies conducted with extracts of *G. elegans*, a raw material for NAOs, showed improvement in stool symptom score on the PAC-SYM and abdominal discomfort scores [11]. Therefore, future research requires expanded clinical studies to evaluate the effects of NAOs on obesity and gut health. Finally, seaweed intake was not restricted during the study period. Korea, Japan, and parts of China have the highest seaweed consumption [51]. Therefore, even if the daily intake of nutrients was the same, the results of the study could be influenced by the amount of seaweed intake. Therefore, a tight dietary restriction of seaweed is required in the daily diet.

However, our study has a good design and advantages. It can provide substantial evidence for the anti-obesity effect of NAOs.

## Conclusion

As a result of a 16-week, randomized, double-blind, placebo-controlled clinical trial on the anti-obesity effect of NAOs in overweight and obese subjects, VFA and VSR were significantly decreased, and weight and BMI were significantly suppressed from increase compared to the placebo group. Therefore, NAOs have a beneficial effect on obesity prevention, suggesting that they could be developed as an anti-obesity supplement without side effects.

## Data Availability

The datasets generated and/or analyzed during the study are not publicly available to study subject confidentiality but are available from the corresponding author on reasonable request.

## Abbreviations

AEs: adverse events
ALT: alanine transaminase
AST: aspartate transaminase
BFM: body fat mass
BMI: body mass index
BUN: blood urea nitrogen
CAN-pro: computer-Aided Nutritional analysis program
CK: creatine kinase
CVD: cardiovascular disease
CRIS: clinical Research Information Service
CT: computed tomography
DBP: diastolic blood pressure
DEXA: dual-energy X-ray absorptiometry
gamma-GT: gamma-glutamyl transferase
GPAQ: global physical activity questionnaire
hs-CRP: high sensitivity C-reactive protein
HDL-C: high-density lipoprotein-cholesterol
HC: hip circumference
HFF: health functional food
ICH GCP: international Conference on Harmonization
LBM: lean body mass
LD: lactate dehy-drogenase
LDL-C: lipopro-tein-cholesterol
MFDS: ministry of Food and Drug Safety of Korea
NAOs: neoagaro-oligosaccharides
PBF: percent body fat
RBC: red blood cell
SBP: systolic blood pressure
SFA: subcutaneous fat area
TFA: total abdominal fat area
T2DM: type 2 diabetes mellitus
VFA: visceral fat area
VSR: visceral-subcutaneous fat area ratio
WBC: white blood cell
WC: waist circumference
WHR: waist-hip ratio
WHO: world Health Organization
